# Supplementary material for: A fault-intrusion-tolerant system and deadline-aware algorithm for scheduling scientific workflow in the cloud
Source: PeerJ Comput Sci. 2021 Nov 2;7:e747. doi: 10.7717/peerj-cs.747 (PMC8576554; doi:10.7717/peerj-cs.747)
Supplement: Supplemental Information 6 [file peerj-cs-07-747-s006.zip › Myproject1.1/docs/org/cloudbus/cloudsim/network/datacenter/package-use.html]

Uses of Package org.cloudbus.cloudsim.network.datacenter


JavaScript is disabled on your browser.


- Overview
- Package
- Class
- Use
- Tree
- Deprecated
- Index
- Help

- Prev
- Next

- Frames
- No Frames

- All Classes

# Uses of Package org.cloudbus.cloudsim.network.datacenter

- Packages that use org.cloudbus.cloudsim.network.datacenter

  | Package | Description |
  | --- | --- |
  |  |  |
  | --- | --- |
  | org.cloudbus.cloudsim.examples.network.datacenter |  |
  | org.cloudbus.cloudsim.network.datacenter |  |
- Classes in org.cloudbus.cloudsim.network.datacenter used by org.cloudbus.cloudsim.examples.network.datacenter

  Class and Description

  AppCloudlet

  AppCloudlet class represents an application which user submit for execution within datacenter.
- Classes in org.cloudbus.cloudsim.network.datacenter used by org.cloudbus.cloudsim.network.datacenter

  Class and Description

  AppCloudlet

  AppCloudlet class represents an application which user submit for execution within datacenter.

  HostPacket

  HostPacket represents the packet that travels through the virtual network with a Host.

  NetworkCloudlet

  NetworkCloudlet class extends Cloudlet to support simulation of complex applications.

  NetworkDatacenter

  NetworkDatacenter class is a Datacenter whose hostList are virtualized and networked.

  NetworkHost

  NetworkHost class extends Host to support simulation of networked datacenters.

  NetworkPacket

  NewtorkPacket represents the packet which travel from one server to another.

  NetworkVm

  NetworkVm class extends Vm to support simulation of networked datacenters.

  Switch

  TaskStage

  Taskstage represents various stages a networkCloudlet can have during execution.

- Overview
- Package
- Class
- Use
- Tree
- Deprecated
- Index
- Help

- Prev
- Next

- Frames
- No Frames

- All Classes
